# A Tale of Two Forests: Addressing Postnuclear Radiation at Chernobyl and Fukushima

**DOI:** 10.1289/ehp.121-a78

**Published:** 2013-03-01

**Authors:** Winifred A. Bird, Jane Braxton Little

**Affiliations:** **Winifred A. Bird** is a freelance journalist living in Nagano, Japan. Her work has appeared in the *Japan Times*, *Science*, *Yale Environment 360*, *Dwell*, and other publications.; **Jane Braxton Little** writes about science and natural resource issues from California’s Sierra Nevada. Her work has appeared in *Scientific American*, *American Forests*, the *Los Angeles Times*, and *Audubon*, where she is a contributing editor.

In a narrow wooded valley just inside the Fukushima evacuation zone, a cold mountain dusk is falling over the terraced plots where Genkatsu Kanno grew rice and vegetables for most of his life. The idle fields are illuminated by lights from his house, where several men bend intently over a low wooden table as they pore over satellite photographs and contour maps.

“So where did you say the drinking water spring is?” asks Tatsuaki Kobayashi, a restoration ecologist at Chiba University, as he studies a print showing the valley’s forest-and-field patchwork. Kanno extends a thick brown finger, carefully tracing the path of the water from its upslope source down to the house that he is permitted to visit but no longer live in. Akihiko Kondoh, a hydrologist also at Chiba University, says the spring could be contaminated with radioactive cesium if heavy rains flood the area.^1^ Kanno, 65, says he’s thinking of digging a well so he can live and farm in the valley again one day.

**Figure:**
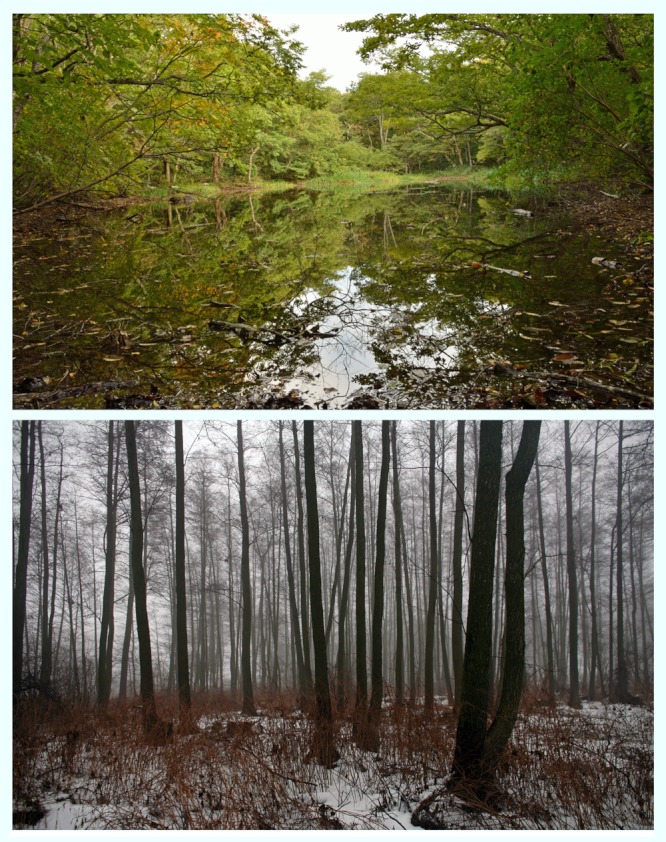
Top: Kawauchi, Fukushima Prefecture 2012; Bottom: Chernobyl, Ukraine 2006 © Winifred A. Bird; © Donald Weber/VII/Corbis

On this evening a year and eight months after multiple explosions at the Fukushima Daiichi Nuclear Power Plant, the men are grappling head-on with one of the most widespread and complex environmental health threats Japan has ever faced: Before fallout released by the March 2011 explosions arrived in the cities that line Fukushima Prefecture’s central corridor, it drifted northwest over the small, cultivated valleys, meandering creeks, and post-and-beam farmhouses of the Abukuma Mountains.^2^ The region’s residents depended on this land for clean water, wild foods, and firewood. Forests and wooded neighborhoods like Kanno’s are at the center of the dilemma.

The questions Kanno and his neighbors are asking about their forests and their families’ health resurface again and again at local, prefectural, and national meetings. They aren’t alone. Around the world, government officials and scientists have been struggling for decades to manage nuclear-contaminated forests in ways that minimize radiation exposures for human populations.

Although significant environmental contamination from accidents at reactors and military facilities dates back to the 1950s,^3^ the dilemma of how to manage contaminated forests emerged most dramatically and most publically after a reactor at the V.I. Lenin Nuclear Power Plant near Chernobyl blew up on 26 April 1986. The accident released a massive amount of radioactive contamination through the western Soviet Union and across northern Europe.^4^^,^^5^ It fell most heavily near the power plant, in a region covered in forests and fields.

The problems the contaminants brought would not disappear quickly. Although radiation from iodine-131 falls by half in just eight days, the half-life of cesium-137 is 30 years; for plutonium-239 it’s 24,100 years. Soviet officials took immediate steps to limit the health impacts of the contamination by removing the region’s residents. Since the 1991 breakup of the Soviet Union, the land has been managed as a protective buffer where trees and other plants help stabilize the contamination within a mostly uninhabited area.

This strategy has become the world’s principal model for handling severe radioactive contamination at the landscape level. For it to work, however, governments must permanently ban people from large areas or accept that those who remain will be exposed to more radiation than the International Commission for Radiological Protection recommends for the general population.^6^

In contrast, Japan’s current recovery plan revolves around removing contamination from the landscape to allow residents to move back home. In this context, contaminated forests represent not a buffer but a threat to public health.

Still, the question of whether forests can—or should—be cleaned up remains extremely controversial. Two years after the Fukushima disaster, Japan’s government has not yet decided whether it will follow the Chernobyl template for forest management or instead try to create a new model for postnuclear environmental remediation.

## The Chernobyl Disaster

Soviet officials began evacuating residents near the Chernobyl power plant a day after the Number 4 reactor exploded. By 1990 more than 350,000 people had been removed and resettled from the most severely contaminated areas of Belarus, Russia, and Ukraine.^7^ This left a 2,600-km^2^ area, now known as the Chernobyl Exclusion Zone, empty of all but the emergency workers drafted to clean up the contamination and those who continued to manage the remaining three reactors, the last of which closed in December 2000. North of the border with Ukraine, Belarus administers the Polesie State Radiation Ecological Reserve, a 2,160-km^2^ restricted zone.

Chernobyl residents were forced to evacuate in areas where surface soil concentrations of cesium-137 exceeded 1,480 kBq/m^2^.^8^ Even the first people to be evacuated got an average effective dose of 33 mSv during the 24 hours before they left (the worldwide average dose equivalent due to natural background radiation has been estimated at 2.4 mSv/year).^5^ The highest doses—in the hundreds of millisieverts—were to the earliest emergency workers, 134 of whom developed acute radiation sickness. ^5^

During the evacuation process residents both within and outside the exclusion zone continued to drink milk and eat locally grown foods laden with iodine-131, which contributed to a dramatic increase in thyroid cancer. ^5^ In the first few weeks after the accident, residents as far away as Kiev feared high levels of iodine-131 would contaminate drinking water, ^5^ although Valery Kashparov, director of the Ukrainian Institute of Agricultural Radiology, says such concerns were never realized.

The number of deaths since then is uncertain due, in part, to the difficulty of distinguishing radiation-caused cancers from others. The Chernobyl Forum, a group of United Nations agencies formed in 2003 to assess the effects of the Chernobyl accident, estimated that 4,000 people eventually will have died from cancer as a direct result of Chernobyl radiation. ^5^ Other estimates have ranged to well over 1 million.^9^

Scientists don’t know exactly what role forest and meadow environments played in mediating human exposures. What they do know is that thousands of hectares of this largely rural area were severely contaminated as a result of the accident. Forests and fields were subjected to a dense cloud of radioactive dust that included cesium-137, strontium-90, multiple isotopes of plutonium, and more than a dozen other radionuclides.^10^

After the accident the Soviet government took steps to reduce long-term radiation exposure originating in these contaminated areas. Among the tasks of some 600,000 cleanup workers known as “liquidators” was felling, bulldozing, and burying all the trees in a 4-km^2^ stand of Scots pines (*Pinus sylvestris*) in the path of the most deadly fallout.^11^ The needles turned cinnamon red before the trees died, and the workers’ nickname for the place, the Red Forest, stuck. Nothing was done to the remaining forests affected by the radiation, says Vasyl I. Yoschenko, head of the radioecological monitoring laboratory at the Ukrainian Institute of Agricultural Radiology. To contain the radionuclides that fell on the zone’s waterways, workers constructed a series of dikes designed to prevent flooding into the Pripyat River, then into the Dnieper River, which flows through Kiev to the Black Sea.^11^ Most of the contamination sank into river and reservoir bottom sediments, where it is relatively stable. ^5^

Throughout the exclusion zone, only the most contaminated areas were treated. The topsoil of some meadows was removed and buried, and buildings in the town of Chernobyl were blasted with sand and washed. Roads were repaved and entire villages bulldozed and buried.^11^^,^^12^ But vast stretches of the contaminated zone were left just as the radiation found them: steel beams dangling in midair from cranes at half-built construction sites, rural homes abandoned, their white-plastered kitchens now occupied by rodents. In the abandoned city of Pripyat, a rusting Ferris wheel holds watch over a deteriorating weed-ridden amusement park.

Gradually, with no one to cut saplings and cultivate farm fields, natural ecological succession began transforming the landscape. The forests that covered 53% of the area before the disaster cover 87% today, according to Yuriy Ivanov, an investigator at the Ukrainian Institute of Agricultural Radiology. Stands dominated by Scots pine have taken over pastures where dairy cattle grazed and farmers grew wheat and flax. Deteriorating dirt roads beyond Pripyat pass through a deceptively lovely panorama: open patches studded with young pines and birch (*Betula pendula*), their leaves golden green, white bark luminescent in soft morning light. Even most pines, more sensitive to radiation than birches,^13^ seem normal.

Despite the passage of 27 years, however, the Chernobyl Exclusion Zone is still one of the most contaminated places on the planet. Levels of cesium-137 in exclusion zone soils vary from around 37 kBq/m^2^ (the threshold for hazardous contamination used by Soviet authorities^14^) to 75,000 kBq/m^2^ in a random pattern that reflects the haphazard releases of radionuclides during the 10-day event.^15^ In the Red Forest, the pines planted after the accident have grown without a central leading stem, rendering them odd-looking dwarfs more like bushes than trees.^13^ Some places are too heavily contaminated to support natural conifer regeneration; pines rarely seed themselves in areas where human dose rates exceed 30 µSv/hr, says Timothy Mousseau, a professor of biological sciences at the University of South Carolina.

Since the initial discharge of radioactive materials, airborne radionuclides have migrated into the forest soil and, for the most part, stayed there. A study of soil contamination in the Red Forest found 90% of the strontium documented in 2001 was located in the top 10 cm of the soil.^16^ Blame—or credit—the forest, says Sergiy Zibtsev, an associate professor of forestry at the National University of Life and Environmental Sciences of Ukraine in Kiev. Trees, grasses, other plants, and fungi trap radionuclides through their basic life cycle: When leaves and needles transpire (release water), the plant draws more water up from the roots. Water-soluble salts of cesium and strontium are chemical analogs of potassium and calcium, respectively, and are taken up in place of these crucial nutrients. In evergreens, Zibtsev explains, the radionuclides gradually accumulate in needles as each season progresses. The needles then fall to the ground, becoming part of the “litter”—the discarded vegetation that covers the forest floor—and returning the radioactive salts to the top layer of the soil in a natural cycle he says takes 10 to 12 years to complete. Without the trees or other permanent groundcover, Zibtsev adds, contaminants would migrate out, blown in dust or carried by water.

People just outside the exclusion zone who depend on forests for work, food, fuel, and other resources pay some of the costs for this environmental service. Many continue to live in areas with cesium-137 soil concentrations greater than 37 kBq/m^2^. They also continue to eat mushrooms, berries, and other local forest foods despite government restrictions and campaigns warning of the dangers.^10^ Mushrooms, the region’s most iconic product, build up especially high concentrations of radioactive cesium.^17^ Cesium-137 content in the majority of edible mushrooms in forest litter decreased by 20–30% between 2005 and 2010. But among species whose feeding networks (mycelia) reach deeper into the soil, the amount of cesium-137 increased during the same period as radionuclides migrated into deeper soil layers.^15^ In 2006 radioactivity in milk still exceeded permissible levels in 40 communities where cows grazed on grass contaminated by cesium-137.^4^^,^^18^

**Figure:**
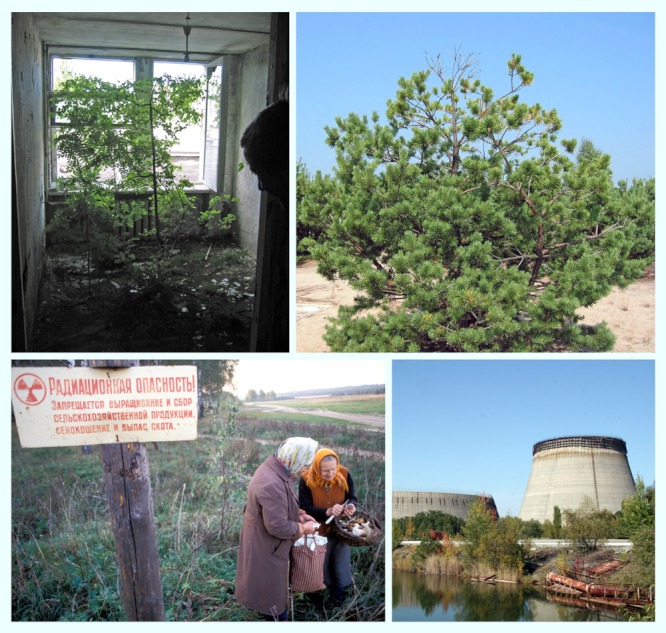
Clockwise from top left: A tree grows from the carpet in a former hotel room in Pripyat, the seed likely transported by wind through the broken window; a 20-year-old Scots pine in the Red Forest shows severe morphological changes resulting from chronic radiation exposure; the unfinished Number 5 and 6 reactors, under construction at the time of the Chernobyl disaster, remain frozen in time, like much of the region; women gather mushrooms near Visokoye, Belarus, under a sign that reads “Radiation danger! Cultivation and harvesting of agricultural crops, haymaking and cattle grazing are prohibited.” Bottom left: © Caroline Penn/Panos. All others: Vasyl I. Yoschenko

Chernobyl contamination is also affecting nonhuman communities. Although the absence of people has attracted a surprising amount of wildlife—moose, wolves, rodents, and birds—their populations are not as diverse or abundant as would be expected in a region where there is little pressure from human communities, says Mousseau.^19^ He and his colleagues have found fewer mammals in high-radiation areas than in less-contaminated areas.^19^ Among birds they have documented reduced longevity and male fertility, smaller brains, and mutations that indicate significant genetic damage compared with the same species in areas of low radiation.^20^

Today the Chernobyl forest and meadow ecosystems are in what scientists call a state of “self repair.” Radionuclides are slowly redistributing themselves in the soils and vegetation through a process expected to continue over many decades, according to a 2006 report by the Ukrainian Ministry of Emergencies.^4^ Ukrainian law requires that the exclusion zone be managed as a barrier that fixes contamination through these natural processes; everything deposited in 1986 must stay within the heavily guarded area. Prohibiting residence and economic activities such as commercial forestry also helps keep contaminated materials from leaving the zone.

Ukrainian officials are convinced they have been successful with their measures to contain fallout from the accident within the exclusion zone. The Number 4 reactor is being converted into an “ecologically safe system” with the construction of a US$2-billion giant arched structure known as a new safe confinement.^4^ Ministry of Emergencies officials believe parts of the mandatory evacuation zone are now safe enough to begin planning for certain activities such as radioactive waste storage and biomass-fueled power plants.^21^

## The Fukushima Disaster

Japan, however, is not yet resigned to either permanently banning residents or exposing them to drastically elevated levels of radiation as a consequence of its own nuclear disaster. Instead, it is attempting to carve a third path forward.

Immediately following the meltdown at the Fukushima plant in March 2011, the Japanese government did evacuate nearby residents. The evacuated area was smaller than that around Chernobyl but far more densely populated, encompassing coastline, farms, and forests in 11 municipalities. At least 157,000 people were either ordered to leave this zone or voluntarily left their homes in other parts of Fukushima.^22^ But by the summer of 2011, the central government had already launched a recovery plan aimed at getting them back.^23^

The strategy centered on extensive decontamination. Isotopes of cesium and other radionuclides were to be removed by early 2014 from houses, roads, farms, public buildings, and wooded areas within 20 m of living areas in all but the most heavily contaminated parts of the exclusion zone (defined as those where the air dose rates for residents could exceed 50 mSv/yr).^24^ The government determined that in the long term this meant getting air dose rates from Fukushima fallout below 1 mSv/yr, although specific targets for 2014 were much more modest.^25^ Some of that reduction would happen through natural decay; Fukushima has a higher ratio of short-lived cesium-134 than areas surrounding Chernobyl.^26^ The rest required hands-on work.

The Japanese Ministry of Environment was put in charge of the project, which has a budget of more than US$6 billion for 2013 alone.^27^ Inside the exclusion zone, the central government was directly responsible for overseeing the work; beyond it, local governments managed the process. Soon contractors and ordinary citizens were hosing down, wiping off, and vacuuming up invisible particles from the surfaces of houses, roads, and schools throughout eastern and central Fukushima, while backhoes scraped soil from fields and stripped grass from parks.^28^ In woodlands near houses, the people raked up leaves and removed lower branches from trees.^29^

**Figure:**
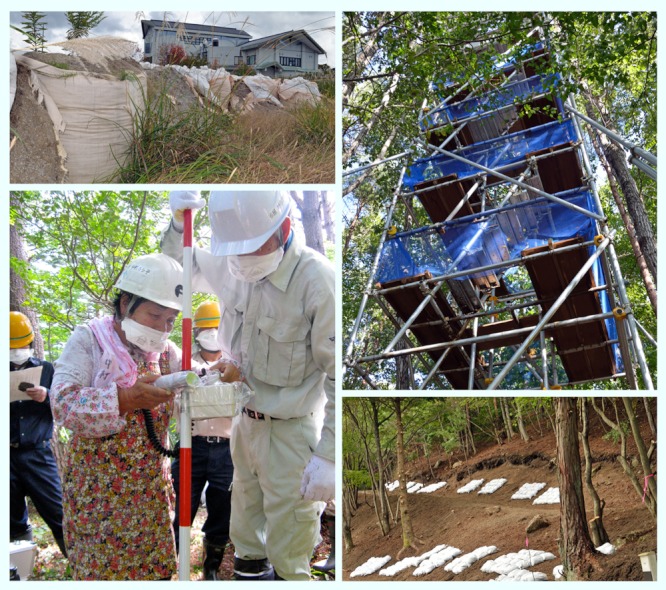
Clockwise from top left: Bags of contaminated soil from Iitate; a tower for monitoring movement of radionuclides in Kawamata; forestry and construction workers join in forest decontamination training at Forest Park Adatara, Otama; trial decontamination behind a home in Kawauchi. All images: Winifred A. Bird

The work continues with mixed success. Radioactive cesium can in some cases be washed or wiped off smooth surfaces like tile, but it easily becomes stuck in the crevices of uneven materials and binds strongly to clay. Decontaminating large areas covered in vegetation, such as parks and gardens, usually means removing and disposing of whatever the cesium is stuck to. Grass and weeds, for instance, are cut, not washed, and dirt is usually removed or deep-plowed, according to Kathryn Higley, head of the Department of Nuclear Engineering and Radiation Health Physics at Oregon State University. The process is labor-intensive, expensive, and prone to corner-cutting.^30^ To make matters worse, rain, wind, animals, and people can move irradiated debris around, recontaminating areas that have already been treated.^31^ As the cleanup proceeded, many Fukushima residents interviewed for this story say they began to suspect that forested slopes were a key source of recontamination—although research has not yet proved this.

For over a year, however, the government remained silent about what should be done in the mixed deciduous forests and evergreen timber plantations that cover the majority of the prefecture near the plant. Finally, in early July 2012, the Ministry of Environment established a committee to discuss forest management.^32^ By the end of the month the group had prepared its initial recommendations.^33^ These proposals will influence final guidelines that determine what happens to forests inside the exclusion zone, where the ministry is directly responsible for cleanup, and define what actions are eligible for subsidies outside the exclusion zone. (As of February 2013 these final guidelines still had not been issued.) The committee concluded there is little need to decontaminate entire forests. It went on to note that removing litter from broad swaths of forest could lead to erosion and undermine tree health, while thinning out trees is unnecessary because it would likely reduce air dose rates only slightly.

The committee based these recommendations on a handful of Japanese government–sponsored studies that indicated only a small percentage of the radionuclides currently in forests is likely to migrate out via water or air.^34^ It also referenced an October 2011 report by an International Atomic Energy Association (IAEA) mission to Fukushima cautioning that overly aggressive decontamination could be extremely costly and generate huge amounts of waste without significantly reducing exposure.^35^ The IAEA report recommended that Japan instead restrict forest and forest product use. It has done so in the case of mushrooms, wild game, and vegetables;^36^ soil amendments and sawdust substrate for mushroom cultivation;^37^ and firewood and charcoal^38^—although, notably, not in the case of timber. Japan’s own guidelines for dealing with the contamination called for prioritizing cleanup in places that would most impact human health.^39^ It was in this context that the ministry committee declared extensive forest decontamination unnecessary.

The backlash from Fukushima was immediate and harsh. One after another, local and prefectural officials and forestry industry representatives attacked the proposal as a city-centric, top-down decision that ignored the deep connections between rural residents and their forested environment as well as the differences between Fukushima and Chernobyl^40^—in northeast Japan, the topography is steep and complex rather than flat; rain is abundant; and forests are closely entwined with densely populated farmland. Although forests have contained the bulk of the contamination around Chernobyl, many doubted they could—or should—play the same role around Fukushima.

Kazuhiro Yoshida, chairman of the Namie town assembly, was among those who traveled to Tokyo to hand-deliver a petition to then–Environment Minister Goshi Hosono calling for extensive forest cleanup. Namie, which is largely forested, lies just northwest of the devastated plant, inside the exclusion zone, and includes some of Japan’s most heavily contaminated land.^2^

“Country life is appealing because we can drink good water and eat wild foods from the mountains. If you put limits on that, you’re not living; you’re surviving,” Yoshida says. He opposes the concept of simply limiting access to contaminated forests. He also fears that contaminant-laden dirt will flow from wooded hillsides into Namie’s rice paddies and reservoirs. Residents won’t be safe unless something is done to reduce the amount of radionuclides in forested areas as well as on farm fields and homes, Yoshida says.

Soil profiles show that within five months after the disaster, between 44% and 84% of radioactive cesium in forest environments was already on the forest floor, most in the litter and top 5 cm of soil.^41^ Anything that causes soil to erode—road building, heavy rains, even decontamination work itself—could carry those contaminants down to the valley floors where human life is centered. Government research has suggested that forests are providing only a small fraction of the radionuclides that are showing up—sometimes at high concentrations—on the bottoms of lakes, in the bodies of river fish, and in rice fields fed by springs in wooded hills.^42^^,43^ In one of the few peer-reviewed studies of this issue to be published so far, investigators compared levels of radiocesium in the water of two small Fukushima rivers to the total estimated radiocesium in the rivers’ watersheds. The authors estimated that during 2011 0.5% of contaminants in one watershed and 0.3% in the other flowed into these rivers, with movement occurring during precipitation and flooding.^1^

Scientists at Japan’s government-funded Forestry and Forest Products Research Institute say they plan to study those long-term patterns. In general, though, Japan has high forest cover and comparatively low erosion rates, says Shinji Kaneko, a soil scientist at the organization, which is closely associated with the Forestry Agency and has become a major research center for irradiated forests. In the long term, the clay soils common in eastern Fukushima may trap more radioactive cesium than the sandy and peat soils around Chernobyl. Kaneko predicts this will lower the rate of transfer to groundwater and wild plants.

Such predictions do not reassure many who live near contaminated forests or are engaged in managing them. Shigeru Watanabe, a prefectural official who oversees forest maintenance in Fukushima, believes that if forests are left alone “people won’t feel safe living in these areas.” He says the prefecture is pushing strongly for extensive decontamination.

Removing litter, branches, or whole trees, however, generates huge quantities of low-level radioactive waste. Fukushima is already struggling to handle millions of cubic meters of contaminated debris from the cleanup.^44^ Stripping the top 5 cm of soil and everything above—litter, fallen branches, trees, and brush—from just the most heavily contaminated forests^45^ would yield another 21 billion kg of debris, according to a study by Forestry and Forest Products Research Institute scientists.^46^ The authors argue that removing just litter is the most efficient approach to decontamination, although it must be done before radioactive particles migrate further into the soil. Litter made up just 3% by weight of the forest components in each sample plot the team analyzed, but as of summer 2011 it contained 22–66% of the radioactive particles in the sample plots.

Prefectural officials want more to be done. A survey by Japan’s Forestry Agency showed that radioactive cesium was split roughly in half between soil and leaf litter on the one hand, and leaves, trunks, and branches on the other.^47^ (In deciduous forests still leafless when the meltdowns occurred, the balance was tilted heavily toward the forest floor.) Watanabe says separate trials conducted by Fukushima Prefecture, which are not publicly available, showed that thinning one-third of the trees reduced radiation by up to 23%, and adding in reductions from removing litter “gets you to about half.” The prefecture plans to begin thinning trees in privately owned forests in 2013 using central government funding, according to Forest Management Department official Norio Ueno.

But the Forestry Agency has found thinning to be about half as effective as the unpublished prefectural trials Watanabe cites.^47^ As time passes, tree removal will likely become even less effective: In Chernobyl, the above-soil portion of trees now holds less than 20% of total forest contaminants, and that percentage is decreasing steadily.^4^

Many of the Fukushima residents interviewed for this article doubt forest decontamination will work; some see the enterprise as a public-relations stunt. Extensive decontamination will, indeed, likely be hard to achieve.^48^ Others in Fukushima suggest that the immense sums being funneled to construction companies managing the cleanup would be better spent on permanently relocating people, including those who live outside the exclusion zone but no longer feel safe in their contaminated neighborhoods. In August 2012 the Ministry of Environment responded to pressure from Fukushima by announcing it would rethink its proposed forest policy. Two months later it announced plans for a working group to consider thinning and clearcutting.^49^

## Two Approaches

Proponents of extensive decontamination see many benefits beyond public safety, according to Ueno: more productive timber plantations (thousands of hectares were in desperate need of thinning even before the disaster), jobs, and, if debris can be burned in biomass power plants, a sustainable energy source. One town actively looking into biomass power generation is Kawauchi, a village deep in the mountains west of the Fukushima Daiichi Nuclear Power Plant. The population of 3,000 has dwindled to 750 since the disaster, according to Kawauchi city hall employee Morie Sanpei. Sanpei, who is in charge of researching the biomass plant, says the town hopes to thin out 50–70% of the trees in the lush forests that loom over small clusters of homes and burn them in a proposed 5,000-kw power plant. In February 2013 Fukushima’s prefectural government also announced plans to build a 12,000-kw biomass power plant that will burn wood from trees thinned in the prefecture’s proposed forest decontamination program.^50^

The Ministry of Environment claims standard filters can keep between 99.44% and 99.99% of radioactive cesium from leaving smokestacks.^51^ These figures are supported by trials at a biomass incinerator in Belarus, conducted as part of the Chernobyl Bio-energy Project, a multiyear international initiative aimed at forest remediation. The researchers involved with that project concluded the health risk from the smoke is “so low that it does not constitute a problem.”They also predicted workers in a biomass plant would receive very little exposure from wood or ash, provided the plant was well designed and work practices well planned.^52^

But Kyoto University nuclear engineer and antinuclear activist Hiroaki Koide believes a proliferation of small biomass plants in Fukushima would be risky; if local officials who lack specialized knowledge are pushed to economize, they might cut corners on critical safety precautions. Indeed, Sanpei notes that cost is a major consideration for Kawauchi. He says that while highly mechanized processing lines reduce plant worker exposure to contaminated materials, they also raise construction costs—possibly beyond what the town can afford.

Using incinerators as a tool to concentrate and contain the Fukushima fallout has the apparent advantage of moving radionuclides out of neighborhoods. But Chernobyl scientists warn that uncontrolled burning of irradiated wood can do the opposite—spread contaminants far beyond their current location. As time progresses in the Chernobyl Exclusion Zone, the natural way trees and other groundcover are trapping radionuclides has developed an ominous downside. The stands that now grow on approximately 1,800 km^2^ are largely unmanaged, according to Zibtsev, the forestry professor. Nikolay Ossienko, part of a forest and fire crew working in the exclusion zone, says he and his coworkers can remove only a few of the dead and dying trees, accomplishing a bare minimum of the thinning required to reduce fire danger and maintain roads for fire vehicle access.

As trees mature and die and more sunlight penetrates the canopy, brush and other undergrowth species are starting to grow in the spaces. The Chernobyl forests are thus developing “fuel ladders” of vegetation that would enable a fire to climb into the tree canopy and jump from treetop to treetop in what’s known as a crown fire.^53^ Without effective forest management, and combined with a general drying trend he attributes to climate change, Zibtsev believes Chernobyl could experience catastrophic fires rivaling those that are being seen with increased frequency in the western United States.^54^ In a low-key conclusion to a 2009 study of vegetation fires in the exclusion zone, Wei Min Hao, an atmospheric chemist with the U.S. Forest Service, and fellow authors said conditions there are “favorable for catastrophic fires.”^53^

The critical difference between those U.S. fires and the potential fires in Chernobyl is that these forests are laden with radionuclides. When they burn, they emit radioactive cesium, strontium, and plutonium^53^ in respirable fine particles, Zibtsev says. Scientists at the Ukrainian Institute of Agricultural Radiology conducted an experimental surface burn on 9,000 m^2^ near the power plant to assess the behavior of the plume and the concentration of radionuclides released in the smoke. The low-intensity ground fire blazed for around 90 minutes, releasing as much as 4% of the cesium-137 and strontium-90 in the aboveground biomass, says Yoschenko. A high-intensity crown fire would release much higher amounts from burning needles, he says. Separate studies have predicted that crown fires in Chernobyl could transport these emissions “hundreds to thousands of kilometers” to human population centers^53^ and, in a worst-case scenario, trigger ongoing government restrictions on contaminated milk, meat, and vegetables.^54^

This is the Chernobyl paradox. “Forests are our friend in health, our enemy when they burn,” says Zibtsev.

Tatsuhiro Ohkubo, a professor of forest ecology at Utsunomiya University, says the risk of forest fires in Japan, especially catastrophic ones, is relatively low compared with Ukraine and limited to a short dry season in spring. Nevertheless, these data present yet another dilemma for Japanese officials and forest residents.

As the sites of the world’s worst nuclear power plant accidents, Japan and Ukraine share the challenge of protecting their citizens even as they hope to return residents to the rural communities where forests sheltered them and provided clean water, food, firewood, and livelihoods. Whether Japan opts for the Chernobyl model, leaving forests to their slow but natural recovery, or chooses to decontaminate them, local residents will inevitably pay a price.

Mizue Nakano, a mother of two who lives in Fukushima City, has seen her teenaged daughters’ health decline. Worried about their exhaustion, bloody noses, and diarrhea, she sent her younger daughter to live with a relative six hours away by car. While stress is a likely cause of these conditions,^55^ Nakano, who remained in Fukushima with her older daughter, is careful to limit her time outside. Bereft of the connection with the forests that surround her city, Nakano is profoundly saddened. “I can’t believe we’ll have to raise our children without taking them out into nature,” she says. Yet decontamination hardly offers a better option: “Even if it were possible to decontaminate the forests, I wouldn’t want to live in the sort of place you’d end up with.”
